# Assessment of the dynamics of inpatient health care delivery in Poland before and throughout the COVID-19 pandemic

**DOI:** 10.1038/s41598-024-62671-2

**Published:** 2024-05-25

**Authors:** Andrzej Śliwczyński, Maciej Jewczak, Kamila Furlepa, Izabela Gołębiak, Adam Rzeźnicki, Michał Marczak, Aneta Ptak-Chmielewska, Paweł Olszewski, Katarzyna Orlewska, Waldemar Wierzba, Ewelina Nojszewska, Ewa Orlewska

**Affiliations:** 1 National Medical Institute of the MSWiA, Warsaw, Poland; 2 Department of Spatial Econometrics, Faculty of Economics and Sociology, University of Lodz, Lodz, Poland; 3 Faculty of Medicine, Lazarski University, Warsaw, Poland; 4 Department of Social Medicine, Medical University of Lodz, Lodz, Poland; 5 Collegium of Management WSB, University of Warsaw, Warsaw, Poland; 6 Warsaw School of Economics, Collegium of Economic Analysis, Institute of Statistics and Demography, Warsaw, Poland; 7 Institute of Political Studies of the Polish Academy of Sciences, Warsaw, Poland; 8 Collegium Medicum Jan Kochanowski University Kielce, Polska, Poland; 9 Department of Applied Economics, Collegium of Management and Finance, Warsaw School of Economics, Warsaw, Poland

**Keywords:** Diseases, Health care, Mathematics and computing

## Abstract

A situation of emergency involving the whole population introduces changes in the dynamics of the health services that are provided. The magnitude of these shifts should be also linked to the medical speciality within which the health benefits are delivered. The aim of the paper was to identify changes in tendencies in inpatient medical service delivery during the COVID-19 pandemic by medical specialties. On the basis of a database extracted for in-patient treatment received from the public payer, a retrospective analysis was carried out. Comparing the values of the dynamics of the services provided in each medical speciality, the period before the COVID-19 pandemic was collated to the years of the pandemic (2020–2021). In the period before COVID-19, positive patient dynamics were observed in more than half of the specialities. Between 2020 and 2021, virtually all specialties reversed the trend and negative dynamics were recorded. The dynamics in 2021 indicate a process of return to the values from 2015 to 2019. Emergency situation has affected the dynamics of healthcare provision in different specialities to various extent. The most resistant to the negative impulses of the state of emergency were the areas that are strictly organisationally and financially defined (e.g. the group of “therapeutic and drug programmes”).

## Introduction

The COVID-19 pandemic has also changed the organisation of healthcare services delivery to patients, with a number of ongoing and published studies reporting significant changes in healthcare utilisation^1–3^. Recorded level of monthly mortality in Poland can be an indicator of these changes and a measure of the severity of the epidemiological situation, which during the pandemic was much higher than the average from the prepandemic years^4^.

In Poland, lockdown-type control measures (suspended classes at schools, or lectures at the universities, remote work at the offices, cancelled mass events or restrictions on social gatherings and bans on unnecessary movement) were implemented on 10–12 March 2020. Restrictions were loosened starting from 20th April 2020. During autumn, the situation deteriorated again, and between 28th December 2020, and 31st January 2021 another lockdown was introduced in Poland. Throughout 2021, the second year of the COVID-19 pandemic, specific occupational health and safety regulations were reintroduced due to increase in infection rate. Subsequent lockdown policies took effect on 20th March 2021 and were valid until 9th April 2021. Restrictions introduced to block the channels of the virus transmission, such as mandatory quarantines or orders to stay at home, also successfully limited patients access to healthcare services, while promoting their provision without direct contact (via telemedicine)^5^. For many patients with chronic or severe illnesses, access to healthcare services was severely hampered or even completely blocked^6^. Examining the impact of organisational health system changes related to reducing COVID-19 transmission arising in healthcare utilisation on population health outcomes and costs introduces significant methodological problems. Regardless of these challenges, quantifying and characterising the changes and their impact on health outcomes and costs can help healthcare systems in Europe and worldwide to optimise the use of resources after a pandemic. It also provides a starting point for preparing healthcare systems to function in emergencies and population-related diseases^7^.

The lack of analyses in this area (studies during this period have mainly focused on assessing the level of covid-19-related morbidity and mortality) warrants research on changes in the dynamics of healthcare services provided per patient and the costs of completed procedures for Polish population. The aim of the analysis was to identify changes in tendencies in inpatient medical service delivery during the COVID-19 pandemic by medical specialties.

## Material and methods

A retrospective analysis of the National Health Fund database was performed (NHF - the only public payer in Poland; database status of NHF as of 29th April 2022) in the area of hospital treatment by obtaining information on individual medical specialties in the years 2015–2021 concerning:

- the number of patients under hospital treatment (HT);
- the values of funds paid to medical entities (under hospital treatment - HT). The source of the data was the official website, which provides ready-made compilations, as well as the possibility of requesting more detailed data: https://ezdrowie.gov.pl/portal/home/badania-i-dane/zdrowe-dane.

In accordance with Polish law, healthcare entities receiving public funding (from the NHF budget) are obliged to report electronically the services provided. These include, among others, patients’ ID (Polish PESEL no.), diagnosis of illness (according to ICD-10 classification), medical procedure performed (according to ICD-9 classification) Data on medical services provided was aggregated at the level of medical specialties. The paper does not constitute a medical experiment within the meaning of Article 21 (1) of the Law on the Profession of Physician and Dentist of 5th December 1996 (Journal of Laws of 2022, item 1731, as amended) and does not require an opinion of the Bioethics Committee at the PIM MSWiA.

The dynamics of changes in the number of patients and the expenditures incurred in the period 2015–2019 (the period before the pandemic) were taken as a reference point in the analyses, and used to assess changes in the system in the pandemic years 2020 and 2021. It was assumed that if the COVID-19 pandemic had no impact on the analysed determinants it should expected that in 2020–2021 the healthcare system would be characterised by a similar rate of dynamics of variables as in the pre-pandemic years (2015–2019), because these processes would reflect demographic changes and consider the epidemiological profile of the population. In order to obtain answers about changes in phenomena over time, the values of chain and single-basis indexes of dynamics were used [Helena Kassyk-Rokicka, Statystyka nie jest trudna. *PWE, Warszawa* (1992)]:

$${i}_{\frac{t}{t-1}}=\frac{{y}_{t}}{{y}_{t-1}}\text{or } {i}_{\frac{t}{{t}_{0}}}=\frac{{y}_{t}}{{y}_{{t}_{0}}},$$

where: *t* is the current time period, *t*–1 is the period 1 unit of time e earlier, *t*_0_ is the time period considered as the base.

To evaluate a longer period of observation, the geometric mean was used (the root of the nth degree of the product of n variables or is the root of the *T* degree from the quotient of the absolute magnitudes of the studied phenomenon in the period/moment last relative to the first). The average rate of change of the phenomena included in the form of time series was calculated using the single-basis approach, according to the formula:

$${\overline{i} }_{g}=\sqrt[T]{\frac{{y}_{t}}{{y}_{t0}}},$$

where: *n* − 1 = *T.* The geometric mean rate of change informs about the average variability of the phenomenon in a rhythm of every period/moment, assuming that the changes run unidirectionally over the entire time span of the sample. The use of the geometric mean allowed us to determine how the variability of the analyzed aggregates proceeded in the precovid period. A reference point (reference value - *RV*) was established for the analyses, defined as:

- the average number of patients and financial value of services in 2015–2019 by the medical specialties;
- the medium-term rate of change in the number of patients and their values according to the medical field.

The benchmark was established by calculating the geometric mean, which allowed estimation of the annual change dynamics recorded in the period of 2015–2019. The use of the geometric mean allowed us to determine how the volatility of the analysed aggregates was conducted during the pre-covid period between 2015 and 2019. Subsequently, correlation analysis was used to verify the convergence of trends, comparing pre-covid with pandemic periods. Thanks to the use of a medium-term rate of change, it was possible to assess the annual trend, which solves the problems of assessing the exact beginning/ending of the pandemic period, or those arising from the way of collecting data (e.g., as of December 31st, or over the entire given time period) the results obtained in this way reproduce a change comparable both in time and in the subject sets.

To enable the conversion and comparison of monetary values, the costs discussed in the article in (Suppl Appendix Table 1) have been recalculated using a conversion factor based on purchasing power parity standard for the year 2021 and expressed in USD^8^.

The 5-year period of providing services before the COVID-19 pandemic, selected as the baseline, allows for a direct assessment of changes in the dynamics of providing medical services in hospital treatment in 2020 and 2021 for the following variables:—the number of patients receiving health services—the financial value of these services.

To assess the degree of convergence of process variability over time, the following were used Spearman’s rank correlation coefficient, which is one of the non-parametric measures of the monotonic statistical relationship between random variables, equivalent of the $\rho$ - Pearson coefficient. It is used to describe the strength of the correlation of two characteristics, when they are measurable, the studied population is small in number, and they are qualitative in nature and can be ordered. It is also used to study the relationship between quantitative characteristics when the number of observations is small, but in this case a normal distribution is no longer required. The $\rho$ Spearman correlation coefficient takes values in the range ⟨−1, 1⟩, where the sign next to the coefficient value indicates the direction of correlation (positive—an increase in the value of one variable defines an increase in the value of the other variable; negative - an increase in the value of one variable defines a decrease in the value of the other). The absolute value of the correlation coefficient determines the strength of the relationship between the variables, where 0 is no relationship while 1 is perfectly correlated. The coefficient is defined:

$${\rho }_{S}=1-\frac{6{\sum_{i=1}^{n}d}_{i}^{2}}{n\left({n}^{2}-1\right)},$$

where *d*_*i*_ = *Rx*_*i*_* − Ry*_*i*_ is the difference between the *i*th rank for the *x* variable and the *i*th rank for the *y* variable.

To verify significant differences in the distributions of variables, the Wilcoxon Test for pairs of observations was used. Wilcoxon test is a non-parametric alternative to the test.

Student’s *t*-test for the case of two equal samples that can be paired. This test is often used to compare data collected before and after an experiment to test whether there has been a statistically significant changes in variables distributions. Unlike the Student’s *t*-test, however, it has no assumptions about the sample distribution. Therefore, it can be used in situations where the assumptions of the Student’s *t*-test are not met. While the Student’s *t*-test tests assume in the null hypothesis the equality of arithmetic means in the corresponding populations, the Wilcoxon test verifies the equality of medians. The method of ranking in the Wilcoxon test is analogous to the determination of the Spearman rank correlation coefficient.

### Limitations of the analysis

The analysed data do not include services dedicated to the prevention and treatment of COVID-19 reported under the type “communicable diseases and emergencies” relegated to separate analysis. The services financed under special pathways of organisation and financing of health services in hospital treatment, i.e.: programmes dealing mainly with medicine technologies—Emergency Access to Drug Technologies (EADT) and integrated and coordinated patient care programmes, were also shifted for separate analysis: “KON-JG”, “KON-BREAST”, “KOS-myocardial infarction” (Suppl Appendix Table 2). The selected data source has already been used for retrospective analyses with respect to health issues^9–11^. Data were extracted and grouped using SQL queries from NHF databases, analyses were developed in Statistica, MS Excel software.

## Results

Table 1 shows the growth rates for the number of patients and the number and value of services provided for three selected time periods: 2015–2019; 2019–2020; 2020–2021. Under the assumption of a one-directional rate of change the average annual change rate was estimated and examined.

**Table 1:** Comparison of the dynamics for the number of patients and value of benefits provided in 2020 and 2021 in relation to values for pre-pandemic period.

Specialty	Patients population dynamics			Value of funding dynamics		
	${\overline{{i }_{g}}}_\frac{2015}{2019}$ (%)	${i}_\frac{2019}{2020}$ (%)	${i}_\frac{2020}{2021}$ (%)	${\overline{{i }_{g}}}_\frac{2015}{2019}$ (%)	${i}_\frac{2019}{2020}$ (%)	${i}_\frac{2020}{2021}$ (%)
Allergology	–0.22	–35.94	21.65	6.03	–29.88	20.41
Anaesthesiology	2.96	–12.24	3.18	5.34	–8.46	1.04
Angiology	–5.18	–15.48	–2.92	–1.09	–11.93	14.16
Audiology	2.34	–36.32	34.13	13.74	–23.50	66.05
Chemotherapy	2.04	–2.41	5.95	4.65	–10.50	–4.25
Paediatric surgery	–2.37	–29.26	15.91	9.19	–16.01	17.16
Thoracic surgery	1.11	–15.21	9.52	6.72	–10.77	10.57
Vascular surgery	–1.17	–19.14	12.10	2.70	–5.26	20.47
General surgery	–1.30	–26.92	10.92	4.68	–16.72	18.40
Oncologic surgery	1.23	–15.26	5.92	9.20	–10.30	6.75
Plastic surgery and paediatric plastic surgery	–2.18	–24.46	14.75	4.02	–1.00	13.26
Maxillofacial surgery and paediatric maxillofacial surgery	–1.51	–22.31	20.14	2.99	–9.91	32.04
Pulmonary and paediatric lung diseases	–1.24	–34.94	4.62	4.48	–26.69	8.27
Internal diseases	–3.78	–29.28	–5.57	3.46	–22.04	5.07
Infectious diseases and paediatric infectious diseases	–3.03	–63.83	–19.33	1.25	–63.41	–0.42
Dermatology and paediatric dermatology	–2.41	–38.68	16.13	0.03	–32.37	16.12
Diabetology and paediatric diabetology	1.33	–37.07	15.90	5.64	–34.40	28.62
Endocrinology and paediatric endocrinology	1.88	–28.50	18.26	6.54	–16.43	19.05
Gastroenterology and paediatric gastroenterology	0.04	–29.19	15.22	5.51	–17.74	23.92
Geriatrics	3.03	–38.15	4.85	10.63	–33.85	26.96
Gynaecological oncology	1.58	–14.19	15.47	8.37	–2.49	14.28
Haematology	4.88	–11.36	14.43	5.72	–2.77	20.46
Immunology and paediatric immunology	2.11	–37.51	53.68	5.47	–5.70	31.54
Cardiac surgery and paediatric cardiac surgery	–1.84	–25.31	13.45	–1.08	–19.58	23.76
Cardiology and paediatric cardiology	0.02	–23.33	11.79	0.23	–11.65	21.08
Nephrology and paediatric nephrology	–0.02	–25.36	11.42	5.96	–19.30	16.27
Neonatology	–2.57	–9.77	–8.11	–0.56	–6.46	–1.74
Neurosurgery and paediatric neurosurgery	2.12	–20.49	16.30	5.06	–8.66	24.61
Neurology and paediatric neurology	–1.25	–26.57	2.53	2.92	–15.34	8.57
Ophthalmology and paediatric ophthalmology	10.36	–31.87	11.55	7.01	–26.08	21.40
Oncology and paediatric haematology	3.57	–23.46	26.36	22.49	–3.20	10.92
Clinical oncology and paediatric clinical oncology	4.08	–10.10	0.20	11.19	6.34	–6.30
Orthopaedics and paediatric orthopaedics	1.66	–21.96	14.06	7.56	–15.55	22.55
Otolaryngology and paediatric otolaryngology	–1.69	–38.15	17.76	2.64	–18.57	29.78
Paediatrics	–1.79	–41.91	34.47	2.94	–35.54	40.81
Obstetrics and gynaecology	–1.78	–19.35	4.10	1.34	–15.83	8.19
Therapeutic and drug programmes	16.44	4.10	10.79	12.68	14.46	13.56
Radiotherapy and brachytherapy	2.97	–7.25	–3.67	8.00	–7.15	1.80
Rheumatology and paediatric rheumatology	0.11	–30.80	13.81	3.67	–24.28	21.31
Highly specialised services and surgery for heart and thoracic aorta defects	0.93	–16.31	11.84	7.20	–10.39	14.33
Clinical toxicology	–5.60	–25.54	–7.15	–1.35	–13.80	10.57
Transplantology and paediatric transplantology	1.11	–22.67	5.80	1.56	–11.57	12.40
Urology and paediatric urology	–0.27	–22.40	11.42	5.67	–14.61	16.43

Between 2015 and 2019, for 23 of the 43 specialities analysed, the medium-term rates of change in patient numbers were positive, with the highest noted (16.44%) for therapeutic and drug programmes. For the remaining 20 specialities, the medium-term rates of change were negative and exceeded −5% in the cases of angiology and clinical toxicology. Only for angiology, cardiothoracic surgery, neonatology and clinical toxicology were there also negative rates of change in the value of billing products performed, but these were lower than those observed for the number of patients. Between 2019 and 2020, the rates of change in the number of patients became negative for nearly all of the analysed specialities with the exception of therapeutic and drug programmes (in which case there was an increase of 4.1% recorded). Apart from therapeutic programmes and clinical oncology, the rates of change were also negative for the value of billing products performed. A negative rate of change in both the number of patients and the value of billing products performed was also observed for 15 of the 41 specialities that had a negative rate of change in the number of billing products performed. It is noteworthy that the highest negative rates of change in the number of patients and value of billing products performed were observed in infectious diseases and infectious diseases of children specialities.

In per-patient terms, the average value of billing products increased from PLN 7,937.76 for 2015–2019 to 10,557.81 in 2021, corresponding to an annual increase in the level of expenditures of 4.16% per patient. In contrast, the average value of billing products decreased from PLN 1,981.91 for 2015–2019 to PLN 137.30 in 2021, accounting for an annual decrease in the expenditures of 31.71% per billing product.

**Figure 1:**
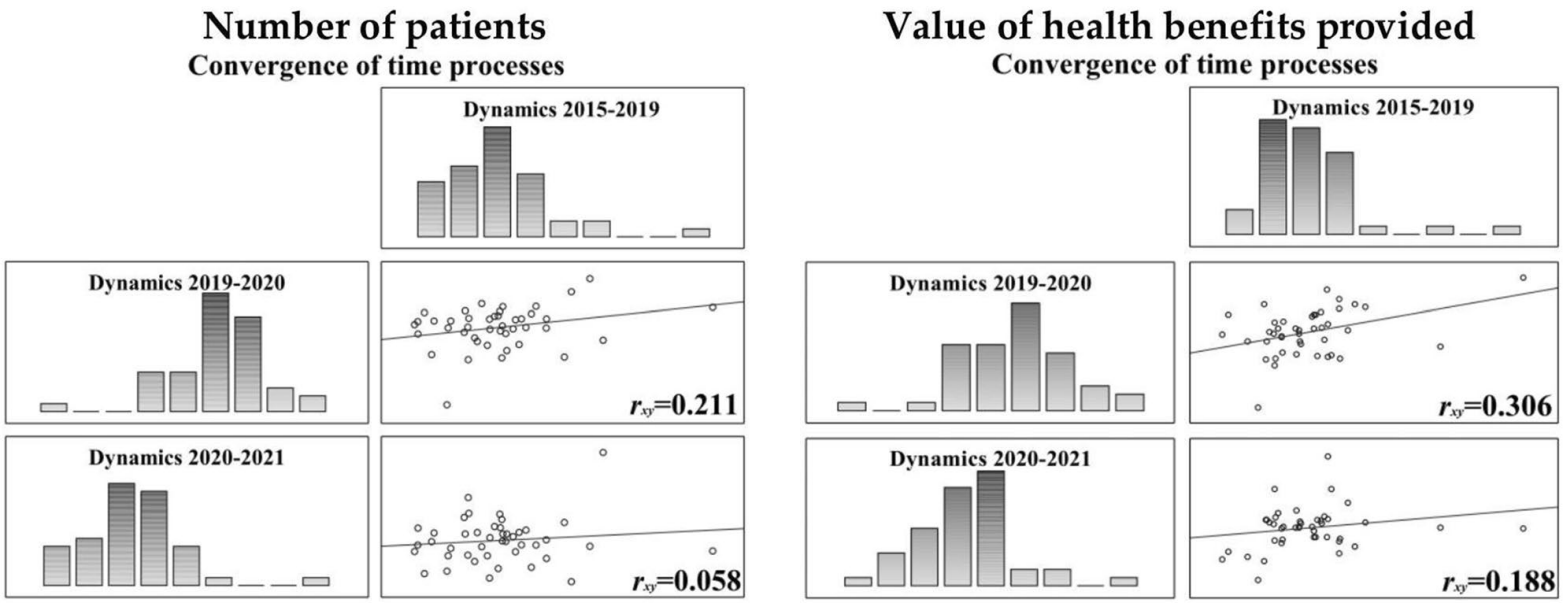
The assessment of dynamics convergence.

The differences in the number of patients and the value of services provided in the pre- and covid periods are very distinctive. Comparing the course of the processes with the adopted reference point, it can be concluded that none of the covid periods showed any convergence. This can be summarised both by the arrangement of the variables in the histograms, as well as from the convergence *r*_*xy*_ Spearman’s coefficients (Fig. 1).

In most fields, a further decrease in the number of patients is to be expected. This would support the conclusion that the backlog after COVID-19 pandemic restrictions cannot be quickly recovered. The analysis indicates that positive indicators of dynamics patient change rates can be expected for immunology and paediatric immunology (+ 11.45%), while the highest negative rates can be expected for infectious diseases and paediatric infectious diseases (−31.64%). In the case of infectious diseases and childhood infectious diseases in 2022, a negative rate of change (−22.14%). Increasing rates of change in the value of services provided are expected in most of the fields.

A gradual return to pre-pandemic trends is expected past 2022 onwards, although given current tendencies in the intensity of change and the health system’s ability to make up for the “losses” the return to the dynamics observed in the years 2015–2019 can be expected in the next 5 years or so. For the number of patients, although there was also a decline in the number of patients treated for some specialties in the pre-pandemic period, it was only during the pandemic that the tendencies in the dynamics of the number of patients and the value of services provided changed, to the point where the statistical concordance of the processes for the population can only be estimated at 20–39%. Looking at changes in the value of benefits performed per patient and on average per service: the mean value of services increased from PLN 7937.76 for the years 2015–2019 to PLN 10,557.81 in 2021, which corresponds to an increase in expenditure of 4.16% per patient annually between 2015 and 2021. The increase in the amount of money spent on services per patient would have to be regarded as a positive development. With the regard to the number of services provided, an upward trend in absolute levels was identified. The mean value of benefits per service decreased from PLN 1981.91 in the period of 2015–2019 to PLN 137.30 in 2021. The more important question, however, is whether the observed changes as a result of pandemic were significant for medical specialties (Table 2).

**Table 2:** Results of Wilcoxon tests.

Null hypothesis			*W* test stat	Sign	Decision
The median differences equals 0 between					
Pop *d*_15–19_	&	Pop *d*_19–20_	−5.68	0.00	Reject the *H*_0_
Pop *d*_15–19_		Pop *d*_20–21_	4.73	0.00	Reject the *H*_0_
val *d*_15–19_		val *d*_19–20_	−5.67	0.00	Reject the *H*_0_
val *d*_15–19_		val *d*_20–21_	4.55	0.00	Reject the *H*_0_

The significance level is *p* = 0.05.

Comparing the results obtained, in each of the analyzed categories (number of patients, value of services for medical specialties). The null hypothesis of distribution convergence of rates of change should be rejected in favor of the alternative hypothesis in each case. This means that the pandemic significantly affected the changes observed in medical specialties.

## Discussion

Healthcare systems around the world have been facing further challenges determined by an increasing demand for patient care (not necessarily those with/after COVID19). Combined with subjective factors (fear, disinformation, telemedicine and mobility limitations), channels of healthcare delivery for all beneficiaries are being disrupted^12–17^. When healthcare systems are overwhelmed, as was the case with pandemic, and the society does not have an access to the care they need, the direct costs of care as well as the unquantifiable results such as observed mortality would increase. The analysis of the number of hospitalised patients and the value of delivered hospital health services in the years 2020 and 2021 compared to the pre-covid period 2015–2019 in Poland showed a significant impact of the COVID-19 pandemic on the indices of care of non-covid patients.

The largest decrease in the number of hospitalised patients was observed in 2020, during the first wave of the pandemic, when strict epidemiologic restrictions have been introduced. This decline was not consistent in all medical fields and may have been caused by a variety of reasons. A striking reduction in hospitalisations due to infectious diseases other than COVID-19 may be partly explained by a reduction in demand and the disruption of transmission channels for all infectious diseases. The disruption in specialised care was rather due to the fact that many hospitals were converted into COVID-19 treatment centres^18^. Therefore, the prioritisation of COVID-19 care reduced the availability of hospital beds, medical resources and technology for non-COVID-19 services^18^. For other fields, in which the largest decline in numbers was observed (e.g. otolaryngology, dermatology, paediatrics, allergology, geriatrics, internal medicine, diabetology), fear of contagion (diabetes, cardiovascular disease are risk factors for severe COVID-19), increased hospitalisation thresholds, the transfer of healthcare or hospital professionals to COVID-19 care and prevention, and barriers imposed by COVID-19-related epidemiological strictures may have contributed to the reduction in healthcare. It is noteworthy, that the decrease in the value of services in these fields was less significant than the decline in the number of patients, which may indicate that more severe cases were admitted^18^. More than 20% drop in the number of patients in fields of transplantology, cardiac surgery, cardiology, paediatric surgery, general surgery, maxillofacial surgery, was probably due to the cancellation of elective procedures during each wave of COVID-19 and patients avoiding or delaying seeking care due to the restrictions of being forced to stay at home^19^.

The impact of COVID-19 on oncology care varied: the largest reductions were seen in oncology surgery, gynaecological oncology, paediatric oncology and haematology; a relatively small decrease was seen in radiotherapy and brachytherapy, but there was an increase in chemotherapy. Thus, it can be assumed that chemotherapy treatment was provided without delay for patients diagnosed before and during the pandemic, while surgical treatment was postponed and, since in these cases the decrease in the number of patients was accompanied by an increase in the value of services, it is likely that hospital treatment was provided to patients with a more severe course or more advanced condition^20^. A significant increase in the number of patients and an growth in the value of services during the pandemic period is characterised by treatment programmes, con-firming that well-organised care, well-defined indications and patient motivation are crucial to ensure access to services under all conditions.

Although the subsequent waves of COVID-19 were more extensive than the initial, their impact on healthcare use in the second year of the pandemic was less significant. This may have been a result partly due to the fact that healthcare workers had already learnt how to cope with the pandemic situation, and even the patients themselves were more able to access medical care after the introduction of vaccination and the reduction of pandemic restrictions. Nevertheless, our forecast for the period following the pandemic indicates that a return of the health care system to its status in 2015–2019 will most likely only be possible in the next 5 years. Our study also confirmed that the organisational, labour resources and financial determinants of the healthcare system were related to the number of patients in the system in the years 2020–2021. This demonstrates the importance of preparing the healthcare system for the future in such a way that the redistribution of resources in a crisis situation does not impair access to healthcare for remaining beneficiaries.

The impact of COVID-19 on the use of healthcare resources has been evaluated in numerous countries, in various phases of the pandemic and by considering different components of the healthcare system^12–17,21–31^.

A systematic review of the literature, which included 81 studies from 20 countries indicated that compared to the pre-pandemic period, the median reduction in health care consumption by May 2020 was in total of 37% and was highest in terms of outpatient visits (42%), while it was 28% for hospital admissions, 31% for diagnostics and 30% for therapeutic and preventive interventions^32^. Nearly half of the 30 studies also reported on the severity of disease symptoms of healthcare users showed greater reductions in the utilisation among populations with milder or less severe disease^32^. According to the literature review on orthopaedic services alone in the first phase of the pandemic, the number of elective operations, trauma procedures and outpatient visits decreased by 80%, 47% and 63%, respectively^33^. US study showed that COVID-19 decreased access to hip and knee arthroplasties and also worsened pre-existing disparities: most affected were non-English speakers and people without a private insurance were most affected^22^. Similar findings are presented in a study on the use of oncology and cardiology services in Italy in March 2020^34^. For oncology, the number of planned procedures decreased from 3.8 to 2.6 per week, there was a 48.4% reduction in admissions for myocardial infarction, a 32% reduction in PCI procedures and a 39.2% extension of the time from occurrence of symptoms to coronary angiography^34^. According to a 2020 US study, outpatient visits dropped dramatically (by 80% in the first days of the pandemic) and hospitalisations and emergency care visits decreased by 30.2% and 37% respectively, with a fourfold increase in the number of tele-visits. By the end of June 2020, the total number of outpatient visits and tele-visits had returned to pre-pandemic levels^35^. Similar results are reported by another US study, which found that the number of outpatient visits in all selected disease areas showed a rapid decline in the early period of the pandemic compared to the pre-pandemic period^36^. An increase in per capita prescription expenditures was observed in all disease domains during the pandemic compared with the pre-pandemic period, while expenditures on home care and outpatient care decreased^36^. The observed popularisation of telemedicine during the pandemic was supported by regulatory changes that led to an expansion of public funding for such services^37,38^. In Poland, there has been a significant increase in the provision of remote services and medical advice (especially in primary care)^39^. The development of this form of medical service provision has appeared to be very much needed, indicating a potential in re-duction of existing resource shortages (especially human resources). The Japanese study, which covers the period to November 2020^21^ indicate that overall hospitalisations and outpatient visits number decreased by 27% and 22% in May 2020, most notably in paediatrics (60% and 51% respectively). Hospitalisations due to respiratory, cardiovascular and malignant neoplasms decreased by 55%, 32% and 10% respectively, while endoscopic procedures and rehabilitation incidences decreased by more than 30%, whereas for outpatient chemotherapy and chronic dialysis this drop was no higher than 10% or none^21^. Reduction in surgeries, longer waiting time between the onset of symptoms and arrival at the emergency room and increase in in number of complications in elective procedures were reported also in Spain^24,25^, Argentina^26^, United Kingdom^27^ and India^28^.

Individual components of cancer care delivery have been studied in many countries. In the US, the proportion of patients receiving at least 1 of common cancer screening examinations during the first 3 months of the pandemic amounted to only 24.0% of the total receiving at least 1 examination in the 3 months prior to the pandemic^29^. In Scotland, the receipt of scheduled anticancer systemic therapy delivery deceased 28.7% in the first 2 months after the outbreak of the pandemic compared with the 2 months prior to the pandemic, with particularly large reductions for some cancers, including colorectal cancer (43.4%)^30^. Canadian study examined the changes in cancer care during the COVID-19 pandemic across the continuum of care, including screening, imaging, surgical treatment, pathological reporting, systemic treatment, radiation treatment, and psychosocial oncological care^31^. This study demonstrated an overall deficit in the first year of the pandemic, particularly pronounced for cancer screening tests, followed by rebounding trends later in the year.

The COVID-19 pandemic has caused severe but heterogeneous disruptions in the availability of healthcare services worldwide with yet unknown remote health consequences. The differences observed between countries may be caused by the different response of governments to the pandemic, the intensity of restrictive measures for Sars Cov-2 containment in the first months of the pandemic and also the resilience of national healthcare systems to emergencies. This resilience largely depends on the organisation of the healthcare system prior to the pandemic. The strength of our study is a focus on the Polish health care system. Because this system relies on universal, publicly funded health care coverage that provides free medically necessary services for all citizens and permanent residents, there was much less likelihood that the study findings were biased owing to differential access to care between the pre-pandemic and pandemic periods. In addition the estimates of in-patient health care services reductions rely on the experience of utilisation in the 5-year period prior to the pandemic. This long baseline period allowed to establish whether service volumes were trending higher over time. Our results, in general terms, reflect the same trend seen worldwide, reflecting a general reduction in the use of health services, but the impact of COVID-19 varies according to the context analysed. The increase in utilization of chemotherapy services observed during COVID-19 outbreak period in Poland confirms that well-organised care continued to provide timely cancer treatment despite significant pandemic-driven disruption. In addition our study explain the gap in knowledge on whether the services have recovered to pre-pandemic levels of care use and, if yes, in what time.

The results of our study can be used for the ongoing planning and reorganisation of the healthcare system and for the management in emergency situations arising from mass events and future pandemics. The pandemic induced natural experiment of reduced healthcare utilisation is an unprecedented opportunity to learn more about which services represent a lower priority for the population and the healthcare system when rapid redistribution of resources is needed to minimise the mortality rate. Indeed, some of the undelivered health services may represent services that are not necessary. According to some studies, a significant reduction in emergency department visits for non-emergent indications may reflect the possibility of implementing a new care strategy that maximises the popularity of such visits in the future^40,41^. The results of our study are a first step towards a better understanding of this crucial issue. There is an urgent need for the health system to plan and implement compensation measures for missed services, which include amongst other things identifying and addressing any adverse health consequences resulting from reduced availability of healthcare. In order to fully understand the indirect health effects of the pandemic and the factors responsible for the disruption of medical services, further research is needed to monitor non-COVID-19 mortality trends. The extent and impact of the wider use of substitution methods, such as tele-medicine, also requires further research with a long-term time horizon.

To conclude, the impact of the COVID-19 pandemic on the delivery of hospital services in Poland varied depending on the specialty. The reduction in services was not greater than in other European countries, and even no disruption was observed in the delivery of services such as chemotherapy and drug/therapy programmes. The aspect of examining the direct impact of COVID-19 restrictions on the number of health ser-vices provided will only be possible once the reported data has stabilised at the end of 2023. The long-term consequences of the disruption of service delivery on the health of the population are so far unknown, but an accurate assessment of these outcomes in the future and the identification of health policy instruments to mitigate this negative impact is key to preparing a health system.

## Conclusion

- An analysis of the dynamics of healthcare services provided in the period 2015–2021 shows that there is heterogeneity between the specialities with regard to the number of patients receiving healthcare benefits.
- In the pre-COVID-19 period, for more than half of the medical specialities the dynamics showed positive rates, while in the COVID-19 period almost all the values turned out to be negative.
- The only section in which positive dynamics was observed during the pandemic period was the group of “therapeutic and drug programmes”, suggesting that the strictly defined organisational provision of benefits (which is the case in this aggregate) is better prepared systemically for extraordinary situations.
- An increase in the value of billed procedures in the 2020–2021 period was also observed that can be attributed to the need to perform medical procedures under a sanitary regime, which raised the cost of their implementation.
- The general—systematic conclusions are:
  - Healthcare systems in all countries need to take measures to widely implement telemedical systems to compensate for the shortages of human resources (medical professionals) during crises and pandemic periods. At the same time, implemented tele-medical solutions should improve the availability of healthcare services during a normal epidemiologic situation^42^;
  - The current situation in healthcare systems should be carefully monitored and analysed on an ongoing basis in order to counteract further declines in access to healthcare;
  - Additional intensive action by governments is needed to restore and maintain the population’s confidence in the effectiveness of a health system aimed at meeting health needs safely and efficiently and controlling the risk of infection in healthcare facilities.
  - The introduction of a coordinated care model in selected medical specialties would result in increased patients’ safety by enabling more accurate surveillance.

### Supplementary Information

Supplementary Tables.

## Data Availability

The data presented in this study are available on request from the corresponding author.
