# Supplementary material for: Assessment of the dynamics of inpatient health care delivery in Poland before and throughout the COVID-19 pandemic
Source: Sci Rep. 2024 May 25;14:11975. doi: 10.1038/s41598-024-62671-2 (PMC11127990; doi:10.1038/s41598-024-62671-2)
Supplement: Supplementary file 1 — Supplementary Tables. [file 41598_2024_62671_MOESM1_ESM.doc]

**Appendix table 1.** Monetary values in the tables and charts converted using a conversion factor based on purchasing power parity for the year 2021 (1 USD = 1.837 PLN (zł))

| **Specialty** | **mean value of services provided** | | **medium-term rate of change** |
| --- | --- | --- | --- |
|  | **2015-2019** | **2019-2021** | **2015-2021** |
| Allergology | 135,720,566.60 | 139,941,939.50 | 1.09% |
| anaesthesiology | 3,929,863,374.00 | 4,500,245,677.00 | 2.19% |
| angiology | 54,221,138.78 | 53,422,299.92 | -0.64% |
| audiology | 15,685,528.55 | 22,974,879.16 | 13.39% |
| chemotherapy | 2,826,180,564.00 | 2,820,263,275.00 | 0.46% |
| paediatric surgery | 573,432,800.20 | 706,484,721.60 | 5.75% |
| thoracic surgery | 628,064,659.80 | 729,200,027.40 | 4.19% |
| vascular surgery | 1,311,041,527.00 | 1,595,294,473.00 | 4.06% |
| general surgery | 5,105,103,700.00 | 5,715,969,256.00 | 2.85% |
| oncologic surgery | 981,314,511.70 | 1,143,030,920.00 | 5.28% |
| plastic surgery and paediatric plastic surgery | 176,237,842.50 | 220,217,208.90 | 4.64% |
| maxillofacial surgery and paediatric maxillofacial surgery | 244,921,622.20 | 286,066,384.40 | 4.97% |
| lung diseases and paediatric lung diseases | 1,381,918,842.00 | 1,390,003,867.00 | -0.93% |
| internal diseases | 5,189,866,905.00 | 5,254,611,562.00 | -1.05% |
| infectious diseases and paediatric infectious diseases | 594,823,004.10 | 383,132,808.50 | -14.79% |
| dermatology and paediatric dermatology | 240,790,969.80 | 215,193,325.30 | -3.93% |
| diabetology and paediatric diabetology | 100,554,555.30 | 102,935,842.90 | 0.83% |
| endocrinology and paediatric endocrinology | 400,295,708.30 | 453,018,544.20 | 4.22% |
| gastroenterology and paediatric gastroenterology | 610,780,807.90 | 697,020,955.40 | 3.97% |
| geriatrics | 213,791,305.60 | 229,823,562.70 | 3.90% |
| gynaecological oncology | 197,734,599.80 | 250,095,896.90 | 7.42% |
| haematology | 742,258,625.50 | 872,837,788.90 | 6.55% |
| immunology and paediatric immunology | 35,699,763.70 | 47,135,212.20 | 7.40% |
| cardiology and paediatric cardiac surgery | 618,587,192.50 | 610,335,163.40 | -0.80% |
| cardiology and paediatric cardiology | 6,350,172,074.00 | 6,845,629,664.00 | 1.28% |
| nephrology and paediatric nephrology | 441,353,593.40 | 493,164,631.50 | 2.84% |
| neonatology | 2,144,978,565.00 | 2,002,023,032.00 | -1.76% |
| neurology and paediatric neurosurgery | 1,679,475,925.00 | 2,054,512,485.00 | 5.60% |
| neurology and paediatric neurology | 2,474,299,089.00 | 2,647,682,675.00 | 0.52% |
| ophthalmology and paediatric ophthalmology | 2,082,281,321.00 | 2,123,246,377.00 | 2.75% |
| paediatric oncology and haematology | 211,398,463.50 | 325,311,085.70 | 15.85% |
| paediatric oncology and clinical oncology | 416,529,215.60 | 530,870,718.20 | 7.26% |
| orthopaedics and paediatric orthopaedics | 5,376,038,720.00 | 6,258,464,694.00 | 5.58% |
| otolaryngology and paediatric otolaryngology | 1,317,686,710.00 | 1,432,024,167.00 | 2.69% |
| paediatrics | 1,522,458,880.00 | 1,522,446,964.00 | 0.32% |
| obstetrics and gynaecology | 4,359,067,614.00 | 4,240,715,750.00 | -0.67% |
| therapeutic and drug programmes | 7,380,666,515.00 | 10,417,406,202.00 | 13.12% |
| radiotherapy and brachytherapy | 2,232,776,371.00 | 2,468,521,770.00 | 4.28% |
| rheumatology and paediatric rheumatology | 442,152,627.20 | 465,122,560.50 | 0.99% |
| highly specialised services and surgery for heart and thoracic aortic defects | 1,296,186,329.00 | 1,445,375,213.00 | 5.17% |
| clinical toxicology | 78,308,070.61 | 79,136,197.89 | -1.69% |
| transplantology and paediatric transplantology | 521,136,457.60 | 521,258,775.00 | 0.93% |
| urology and paediatric urology | 1,379,721,562.00 | 1,547,030,507.00 | 3.65% |
| Poland | 68,015,580,238.00 | 75,859,201,082.00 | 3.28% |

**Appendix table 2.** Abbreviations and symbols used in the article

| **Abbreviation** | **Expansion** | **Meaning** |
| --- | --- | --- |
| NHF | National Health Fund | National payer for medical services, financing the services from public funds |
| EADT | Emergency Access to Drug Technologies | Separate funding pathway for “last resort medicines |
| KON-JG programme |  | Comprehensive oncological care for patients with colorectal cancer |
| KON-BREAST programme |  | Comprehensive oncological care for patients with breast cancer |
| KOS-myocardial infarction programme |  | Comprehensive care programme for patients with myocardial infarction |
| therapeutic and drug programmes |  | Separate funding pathway for medicines based on their close monitoring |
